# Biomaterials for Guided Tissue Regeneration and Guided Bone Regeneration: A Review

**DOI:** 10.3390/dj13040179

**Published:** 2025-04-21

**Authors:** Nathália Dantas Duarte, Paula Buzo Frigério, Gloria Estefania Amaya Chica, Roberta Okamoto, Rogério Leone Buchaim, Daniela Vieira Buchaim, Michel Reis Messora, João Paulo Mardegan Issa

**Affiliations:** 1 Department of Diagnosis and Surgery, Araçatuba School of Dentistry (FOA-UNESP), São Paulo State University, Araçatuba 16015-050, Brazil; nd.duarte@unesp.br (N.D.D.); paula.frigerio@unesp.br (P.B.F.); 2 Department of Oral and Maxillofacial Surgery and Periodontology, Ribeirão Preto School of Dentistry (FORP-USP), University of São Paulo, Ribeirão Preto 14090-904, Brazil; g.amayachica@usp.br (G.E.A.C.); m.messora@forp.usp.br (M.R.M.); 3 Department of Basic Sciences, Araçatuba School of Dentistry (FOA-UNESP), São Paulo State University, Araçatuba 16015-050, Brazil; roberta.okamoto@unesp.br; 4 Department of Biological Sciences, Bauru School of Dentistry (FOB-USP), University of São Paulo, Bauru 17012-901, Brazil; rogerio@fob.usp.br; 5 Graduate Program in Anatomy of Domestic and Wild Animals, Faculty of Veterinary Medicine and Animal Science, University of São Paulo (FMVZ/USP), São Paulo 05508-270, Brazil; danibuchaim@alumni.usp.br; 6 Anatomy Department, Medical School, University Center of Adamantina (FAI), Adamantina 17800-000, Brazil; 7 Postgraduate Department, Dentistry School, Faculty of the Midwest Paulista (FACOP), Piratininga 17499-010, Brazil; 8 Department of Basic and Oral Biology, Ribeirão Preto School of Dentistry (FORP-USP), University of São Paulo, Ribeirão Preto 14040-904, Brazil

**Keywords:** biocompatible materials, dental implants, guided bone regeneration, guided tissue regeneration, periodontics, tissue engineering

## Abstract

This review aims to provide an overview of the types of membranes, bone substitutes, and mucosal substitutes used for GTR and GBR and briefly explores recent innovations for tissue regeneration and their future perspectives. Since this is a narrative review, no systematic search, meta-analysis, or statistical analysis was conducted. Using biomaterials for GTR and GBR provides a reduction in postoperative morbidity, as it contributes to less invasive clinical procedures, serving as an alternative to autogenous grafts. Moreover, randomized clinical trials (RCTs) and systematic reviews are essential for the evaluation of new biomaterials. These studies provide more robust evidence and help guide clinical practice in the selection of safer and more effective biomaterials, allowing for the personalization of treatment protocols for each patient.

## 1. Introduction

The supporting periodontium consists of the cementum, periodontal ligament, and alveolar bone [1]. Its primary functions include anchoring the tooth within the alveolus, distributing and absorbing masticatory forces, and protecting against periodontal pathogens by isolating the subjacent tissue [2]. In patients with dental implants, the peri-implant tissues differ anatomically due to the absence of cementum and periodontal ligaments [3].

Periodontitis is a chronic inflammatory disease that affects the supporting periodontium [4]. The damage to these tissues results in periodontal defects, which are sequelae of periodontal disease, leading to tooth loss and other complications [5]. In this clinical condition, guided tissue regeneration (GTR) is indicated, which aims at the regeneration of the supporting tissues [6]. The biological principles of GTR involve the use of physical barriers to prevent epithelial and connective tissue cells, which have a higher turnover rate from contacting the root surface during the healing process, allowing for the restoration of the supporting periodontium tissues [7,8]. The first research in this field was introduced by Nyman et al., who utilized a Millipore^®^ filter as a membrane to preserve space and isolate the bone defect around a periodontal tooth from the surrounding soft tissue [9].

Additionally, in the presence of peri-implant defects and atrophic areas, guided bone regeneration (GBR) is essential to create a receiver site with a favorable bone quantity, which is crucial for the success of osseointegration and a good prognosis for oral rehabilitation with dental implants [10]. GBR is derived from GTR, as it follows the same biological principles but with a focus on directing new bone formation to treat bone lesions [11]. The success of GBR is reached through the exclusive repopulation of osteoprogenitor cells [12].

The healing process consists of the formation of an initial clot and inflammatory, proliferative, maturation, and bone tissue remodeling phases [13]. This process can lead to the replacement of the damaged tissue with an unspecific type, known as repair, that results in fibrosis and scar formation [14]. In contrast, regeneration restores the specific damaged tissue, maintaining both macrostructure and function [14]. For this, GTR and GBR utilize biomaterials, which, according to the National Institutes of Health (NIH), are defined as any substance, excluding drugs or combinations of substances, synthetic or natural in origin, that can be used for any period as a treatment, whether total or partial, to enhance or replace any tissue, organ, or body function [15].

Biomaterials are classified based on their origin as autogenous, allogenous, xenogenous, alloplastic, or synthetic [16]. In terms of physical characteristics, they can be inorganic (mineralized) or organic (demineralized) [16]. Additionally, based on their absorption properties, biomaterials are categorized as non-absorbable or absorbable [17]. Due to this variety of biomaterials, this review aims to provide an overview of the types of membranes (barriers), bone substitutes, and mucosal substitutes used for GTR and GBR, presenting their main differences. In this way, it seeks to assist clinicians in selecting the most suitable biomaterial for each clinical case. In addition, this review briefly explores recent innovations for tissue regeneration and their future perspectives.

## 2. Methods

This narrative review was conducted following the guidelines of the Scale for the Assessment of Narrative Review Articles (SANRA) [18]. Electronic searches were carried out in the PubMed and Web of Science databases. The search included the following keywords: absorbable membranes, allogenic mucosal substitute, alloplastic or synthetic bone, autogenous bone, bone substitutes, collagen-based membranes, epithelial and connective tissue gingival graft, guided bone regeneration, guided tissue regeneration, mucosal substitutes, non-absorbable membranes, synthetic membranes, xenogenous bone, xenogenous mucosal substitute, and related terms for each biomaterial described in this review, with no filter or restrictions on time or language. Information was from in vitro and in vivo studies, clinical studies, reviews, and systematic reviews. Due to the large volume of publications in this field, studies were chosen based on their clinical and/or scientific significance. Since this is a narrative review, no systematic search, meta-analysis, or statistical analysis was applied.

## 3. Membranes (Barriers)

Membranes act as a physical barrier, preventing the proliferation of epithelial cells and fibroblasts during regeneration [17]. Additionally, membranes contribute to mechanical stability, ensuring the necessary space for GTR and GBR, allowing for their use as a covering material to keep the other biomaterials used in the position [19]. The ideal properties of membranes are biocompatibility, occlusivity, and selective permeability, as well as the ability to create and maintain space, good integration with soft and hard tissues, and easy handling [9].

### 3.1. Non-Absorbable Membranes

The first generation of membranes consisting of non-absorbable membranes was popular in the 1990s, made from an inert hydrophobic and stable polymer known as expanded polytetrafluoroethylene (e-PTFE), with good biocompatibility and mechanical stability [20]. However, this membrane presents a high risk of premature exposure of 30 to 40%, increasing the probability of infection [21]. Due to this disadvantage, a high-density polytetrafluoroethylene (d-PTFE) was developed [22]. This membrane is non-expandable, has a high-density structure, and is not permeable to bacteria because of low porosity [23]. The d-PTFE has been indicated for use in areas with large ridge atrophies to prevent contamination in case of premature exposure of the membrane [22]. Additionally, a second surgical procedure is required for non-absorbable membrane removal, which can damage the newly formed tissues [17]. A titanium-reinforced microporous membrane (Ti-PTFE) (OpenTex^®^-TR, Purgo Biologics, Seoul, Republic of Korea) offers major support, was tested, and showed good results when used for vertical bone augmentation around dental implants [24]. Pure titanium meshes are also an alternative to non-absorbable membranes, as they have properties of elasticity, stability, and plasticity [25].

A current randomized controlled clinical trial compares PTFE membranes, CAD/CAM semi-occlusive titanium meshes, and CAD/CAM occlusive titanium foils for GBR in atrophic arches and highlights the potential advantages associated with the use of different CAD/CAM technologies [26]. In addition, the use of non-absorbable membranes in GBR may represent a viable clinical option, particularly for vertical reconstruction of up to 6 mm in the posterior mandible, but this approach should be performed by experienced surgeons [27]. The choice of using non-absorbable membranes is especially indicated for cases that require stability for an extended period. However, a control protocol is required to avoid contamination and minimize its disadvantages. The commercial PTFE membranes available are presented in Table 1.

### 3.2. Absorbable Membranes

The second generation of membranes is absorbable, and they are classified as collagen-based or synthetic [28]. Their main advantage is that they require only one surgical procedure, reducing morbidity and tissue damage and increasing patient comfort [29]. However, the disadvantage is the unpredictable absorption time and degradation rate, which can affect the predictability of the regenerative procedure [30].

#### 3.2.1. Collagen-Based Membranes

The collagen-based membranes are the most commonly used for GTR and GBR [31]. Collagen is the main protein found in connective tissue; it enhances the osteogenic differentiation of mesenchymal stem cells (MSCs), inhibits osteoclasts during the mineralization process [32], and acts on signals via the integrin or non-integrin receptor cell surface. In addition, it stimulates the migration of fibroblasts, endothelial, and inflammatory cells to the wound site while also minimizing axonal degeneration and disintegration [33]. The collagen-based membrane, presented as type I and III, mainly originated from bovine and porcine, and has benefits such as high biocompatibility, low immunogenicity, hemostatic capacity, chemotactic action on fibroblasts and osteoblasts, and dimensional stability [31]. Its main disadvantages are the high cost, low mechanical strength, and rapid degradation rate [30]. This degradation is influenced by several factors, including proteolytic enzymes [34], inflammatory response [35], bacterial proteases [36], and intrinsic characteristics of the membranes [37]. Collagen is degraded by collagenase and matrix metalloproteinases (MMPs) [38]. After collagen fragmentation by the enzymes, polymorphonuclear leukocytes and macrophages phagocytose the membrane residues as part of the immunoinflammatory response [39]. The proteolytic enzymes of Porphyromonas gingivalis cells may modify the membrane’s physical structure, potentially affecting their biological properties [36]. Therefore, the rapid degradation rate can compromise the success of the regenerative procedure [30]. Moreover, collagen-based membranes can be combined with other biomaterials for necessary support [40].

**Figure 1:**
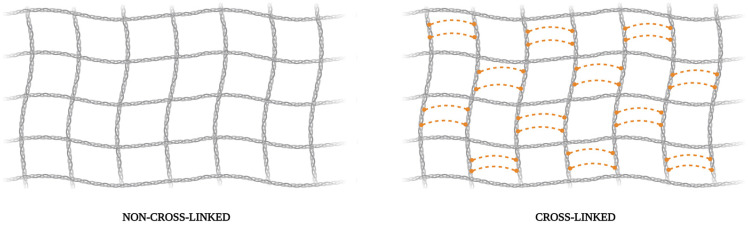
Representative figure illustrating the differences in linkers between collagen fibers of non-cross-linked and cross-linked collagen-based membranes. Created with BioRender.com.

The intrinsic characteristics of collagen membranes include the type of cross-linking present in their composition [41], as shown in Figure 1. Collagen membranes that only contain original links between collagen fibers are known as non-cross-linked membranes, which have faster absorption between 1 and 2 months, greater flexibility but less mechanical resistance, and better biocompatibility [41,42]. In contrast, reticulated membranes, cross-linked, have additional links between collagen fibers developed through a physico-chemical process that makes fibers more resistant to degradation, and the rate slows down after about 3 to 6 months. The strength of collagen fibrin to degradation is associated with the density of molecular cross-links, as it has difficult hydrolytic access [43]. However, they are less biocompatible due to biological integration and reduced angiogenesis [44]. Other factors like porosity, thickness, and weight influence the degradation rate.

The choice of the ideal membrane depends on the clinical case. For small defects and healthy patients, they generally benefit from the use of non-cross-linked membranes. In contrast, larger defects or systemic conditions such as diabetes and osteoporosis may require cross-linked membranes, which offer greater stability and a prolonged barrier function. In these situations, a longer space maintenance period is necessary, as the healing and repair processes are compromised. Moreover, studying membrane degradation in humans is challenging due to ethical reasons. Therefore, well-designed animal studies are recommended to evaluate healing and tissue responses around the membranes, considering different defect sizes and animal models [45]. Table 2 highlights the resorption time and clinical indications of different commercially available collagen membranes.

#### 3.2.2. Synthetic Membranes

The synthetic membranes are produced using biodegradable polymers, mainly aliphatic polyesters, such as polylactic (PLA), polyglycolic (PGA), polycaprolactone (PCL), and polylactic-*co*-glycolic acid (PLGA), as well as polydioxanone (PDO), their blends, and other copolymers [50]. The high rate of manipulation of aliphatic polyesters reduces the availability of the biomaterial, affecting the time of membrane function in the oral cavity, in addition to causing a specific inflammatory response [51]. Additionally, synthetic biomaterials generally do not recognize cell signals, often requiring surface modifications to improve interactions between cells and the biomaterial [51]. Currently, only a limited number of commercially synthetic resorbable membranes are available, and some examples are presented in Table 3, while many others are still undergoing preclinical studies [17].

## 4. Bone Substitutes

**Figure 2:**
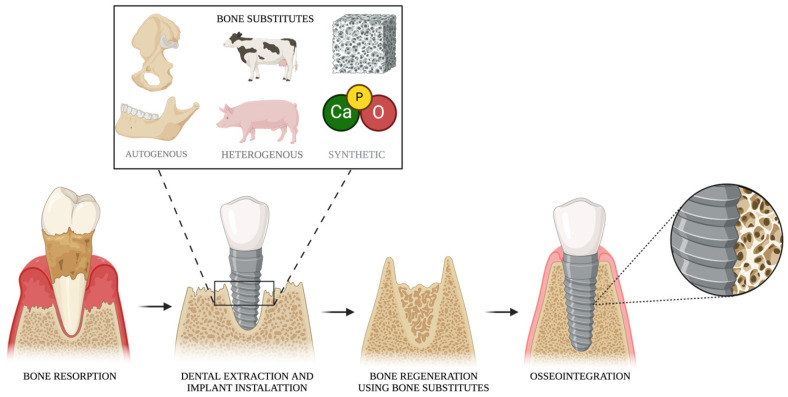
Representative figure illustrating the bone repair process using different types of bone substitutes. Created with BioRender.com.

Bone substitutes are widely used to enhance support and increase bone volume in dental and maxillofacial procedures, including implant placement, sinus augmentation, expansion of the atrophic alveolar ridge, and the treatment of bone defects following dental extractions [54]. Figure 2 illustrates the bone repair process and highlights the different types of bone substitutes. An ideal bone substitute should possess several key properties as follows: biocompatibility, resorbability, osteoconductivity, and osteoinductivity [54]. Additionally, it should closely resemble natural bone in structure, exhibit adequate porosity for cellular integration, provide sufficient mechanical strength, be easy to handle and install, ensure biological safety, and offer a favorable cost–benefit ratio [55].

### 4.1. Autogenous Bone

Autogenous bone grafts are considered the gold standard in reconstructive procedures due to their osteoinductive, osteoconductive, and osteogenic potential [56,57]. These grafts release essential growth factors and cytokines that regulate the activity of osteoblasts and osteoclasts, playing a crucial role in bone regeneration [58]. Among these, bone morphogenetic proteins (BMPs) stand out as key growth factors responsible for the proliferation and differentiation of mesenchymal progenitor cells into osteogenic cells, a process known as osteoinduction [58]. Simultaneously, autogenous grafts serve as osteoconductive scaffolds, providing a structural framework for angiogenesis, which enhances osteoblast nutrition and supports new bone formation [59]. Additionally, they exhibit osteogenic potential by transporting mesenchymal progenitor cells that differentiate into osteoblasts, further contributing to bone regeneration [60].

In clinical practice, the most commonly used extraoral donor site is the iliac crest due to its high bone volume and cellular content [61]. However, intraoral donor sites are frequently utilized, including the maxillary tuberosity [62], the chin (menton) [63], and the retromolar area [64]. In addition, autogenous grafts are often obtained from healing extraction sockets, as these sites showed a potential for higher demand for BMPs, optimizing regenerative outcomes [65]. One of the primary advantages of autogenous bone grafts is the absence of immunological rejection or disease transmission risks, making them a biologically safe option [65]. However, their use is limited by factors such as graft availability, increased morbidity, and the complexity of the procedure, as it requires a secondary surgical site for graft harvesting [66]. Recent studies indicate that particulate autogenous grafts are preferable to block grafts, as they provide a greater surface area for osteogenic cell interaction, facilitating more effective bone regeneration [66,67].

### 4.2. Heterogenous Bone Substitutes

Heterogeneous bone substitutes, also called xenogenous, are inorganic deproteinized biomaterials derived from porcine, equine, or bovine sources which undergo thermal or chemical processing to remove organic components that could trigger immunogenic reactions [68]. This rigorous treatment ensures biocompatibility while preserving the inorganic phase, primarily composed of hydroxyapatite [56,69]. The structural integrity of hydroxyapatite maintains the microarchitecture of natural bone, closely resembling human bone tissue [70]. This characteristic is fundamental to the high osteoconductive potential of heterogeneous bone substitutes, as it facilitates chemotaxis and the adhesion of osteoblasts onto its surface, promoting new bone formation [71]. The osteoconductive properties of these materials make them serve as a scaffold for cell migration, vascular infiltration, and subsequent bone formation [72]. The porosity and interconnectivity of the material allow for integration within the recipient site, supporting bone remodeling processes [73]. One of the key advantages of heterogeneous bone substitutes is their reduced surgical morbidity, as they eliminate the need for a secondary donor site, unlike autogenous grafts [66,74]. Additionally, these substitutes contribute to shorter surgical times, improved patient comfort, and greater availability, ensuring consistent and standardized material for clinical applications [66].

However, the heterogenous bone substitute does not have osteogenic and osteoinductive properties and has a slower resorption rate that maintains volume stability, but it can also delay the replacement of the graft by newly formed bone [75,76]. The efficacy of heterogeneous bone substitutes is highly dependent on the processing techniques employed during their production. Differences in deproteinization, sterilization, and sintering temperatures can significantly alter the porosity, surface roughness, and biological performance of the material [77]. In addition, the heterogenous bone substitute has lower mechanical strength compared to autogenous or alloplastic grafts, which may necessitate the use of combinations with other materials for fixation and enhanced stability [78]. The chemical or thermal processing and sterilization protocols minimize the risk of disease transmission, which historically has raised concerns about prion contamination, the disease known as bovine spongiform encephalopathy (BSE) [79]. However, bovine bone substitutes undergo rigorous inspection following local health guidelines to be marketed. For example, Bio-Oss^®^ (Geistlich Pharma, Wolhusen, Switzerland), of Australian bovine origin, is the most common bone substitute used worldwide and complies with the European Union safety guidelines regarding BSE [80]. Most studies indicate that Bio-Oss^®^ is a safe material [80,81]. On the other hand, a systematic review suggests that bovine-derived graft biomaterials, including Bio-Oss^®^, may have a risk of prion transmission to patients [82]. Additionally, a case series highlighted that clinicians should be aware of the potential complications associated with bovine-derived graft materials. The long-term safety of xenografts and their potential association with prion are valid concerns [83]. Commercial examples with reabsorption rates are presented in Table 4.

### 4.3. Synthetic Bone Substitutes

Synthetic or alloplastic bone substitutes are free of immunogenicity and antigenicity because they are free of biological material [88] and have major cultural and religious accessibility, since many patients have cultural and religious restrictions [89,90]. For example, in Turkey, a predominantly Islamic country, animal-derived biomaterials were the least required [91]. The alloplastic bone substitutes are biocompatible, osteoconductive, either degradable or non-degradable, and widely available since they can be produced on a large scale with a controlled composition [88,92] (Table 5). This fact suggests that the development of synthetic biomaterials is a global trend.

This class of bone substitutes includes many types of synthetically manufactured materials, such as bioactive glass, bioceramics, calcium sulfate, calcium phosphate, hydroxyapatites, and polymers [93,94]. All synthetic bone substitutes are derived from calcium phosphate apatites (CaPO_4_), which are the main inorganic component of mineralized tissues [95]. These compounds used for bone regeneration belong to the group of orthophosphates, which includes beta-tricalcium phosphate (β-TCP) and hydroxyapatites (HA), making them bioactive as they react with biological tissues, which is why they are called bioceramics [96]. However, it has been indicated that these two bioceramics exhibit different resorption rates, characterized by different dissolution properties [97]. Thus, β-TCP has a faster resorption rate than HA [97]. Patients with osteoporosis and aged individuals experience impaired healing and bone formation [98,99]. Therefore, a slower resorption rate is desirable so that the biomaterial remains in the body for a longer period, serving as a scaffold for new bone formation. Various combinations of β-TCP and HA in different ratios are available on the market. In such cases, it is recommended that the clinician select a biomaterial with a higher proportion of HA compared to β-TCP.

Bioactive glasses and bioceramics are inert and translucent materials derived from silica, with nomenclature that varies according to their structure and chemical composition [100]. True glasses are amorphous, while bioactive glasses contain less silicate and more phosphate and calcium, which facilitates dissolution in biological tissues [101]. Clinically, bioactive glass is slowly absorbed, resulting in an ionic exchange [102], where phosphate ions regulate osteoblast apoptosis, osteopontin, and the mineralization rate, while calcium ions affect osteoblast proliferation [103]. Calcium phosphate forms on the outer layer of bioactive glass particles in the form of carbonated hydroxyapatite (HCA), structurally similar to bone hydroxyapatite, allowing for interaction between the particle and bone [104]. After HCA formation, phagocytic macrophages are activated, cells differentiate in osteoblasts, and a new bone matrix is secreted [104]. A unique characteristic of bioactive glasses is osteostimulation, a property distinct from osteoinduction and osteoconduction, which stimulates the deposition of new bone inside the internal chambers of bioactive glass particles, where this bone has no connection with external bone [105].

**Table 5:** Commercially available synthetic bone substitutes.

Commercial Name	Manufacturer	Composition	Reabsorption Rate	Reference
Perioglas^®^	NovaBone Products	Bioactive glass SiO_2_ (45%)/Na_2_O (24.5%) CaO (24.5%)/P_2_O_5_ (6%)	6–12 months	[106]
Biogran^®^	Zimmer Biomet	Bioactive glass SiO_2_ (45%)/Na_2_O (24.5%) CaO (24.5%)/P_2_O_5_ (6%)	6 months to 2 years	[107]
BoneCeramic^®^	Straumann	60% HA/40% β-TCP	6 months to 2 years	[108]
MBCP+^®^	Biomatlante	20% HA/80% β-TCP	3–12 months	[109]
Cerasorb^®^ M	Curasan Inc.	100% β-TCP	12 months	[110]

Abbreviations: silicon dioxide (SiO_2_); sodium oxide (Na_2_O); calcium oxide (CaO); diphosphorus pentoxide (P_2_O_5_); hydroxyapatite (HA); beta-tricalcium phosphate (β-TCP).

## 5. Mucosal Substitutes

Mucosal substitutes, also called soft tissue substitutes, are used in periodontal and peri-implant surgeries. They are indicated for increasing keratinized mucosa around teeth and implants, covering gingival recessions, correcting volumetric tissue deficiencies in esthetic zones, and regenerating periodontal defects [111]. They must be able to promote tissue regeneration by stimulating the proliferation of specialized cells, such as fibroblasts, cementblasts, and keratinocytes [111]. Additionally, they must be resistant to early degradation, ensuring stability for a sufficient period to allow for complete regeneration before being resorbed or replaced by new tissue [112].

### 5.1. Autogenous Soft Tissue

The autogenous mucosal substitute, known as a gingival tissue graft, is widely recognized as the gold standard for increasing keratinized gingival tissue, covering gingival recessions, and facilitating peri-implant esthetic surgeries, among others [113]. The free gingival graft (FGG) involves collecting a graft from the palatal mucosa and placing it at the recipient site [113]. It is primarily used to increase the width of the keratinized gingiva and improve tissue stability around teeth and implants [114]. Conversely, a connective tissue graft (CTG) is a subepithelial connective tissue graft obtained from the palate and placed beneath a flap at the recipient site [115]. It is widely used for root coverage, improving gingival thickness, and enhancing esthetic outcomes [113,114]. Lastly, the pedicle graft involves repositioning adjacent gingival tissue to cover a recession defect while maintaining its original blood supply, including both the laterally positioned flap and coronally advanced flap [116].

The advantages of autogenous gingival grafts involve a high success rate, since they ensure excellent biocompatibility and predictable healing, improved tissue integration, long-term stability, and enhanced esthetic outcomes [117]. Despite their benefits, autogenous gingival grafts present some limitations, such as limited tissue availability, surgical complexity, difficulty of removal in the donor site due to high anatomical vascularization of the hard palate, a longer surgical time, and morbidity [118]. However, autogenous gingival tissue grafting remains the best choice for periodontal plastic surgery due to its proven efficacy, biocompatibility, and long-term success [117]. However, alternative mucosal substitutes are continuously being developed to reduce patient morbidity while maintaining favorable clinical outcomes.

### 5.2. Allogenous Mucosal Substitutes

The allogenous mucosal substitute has been an option for periodontal plastic surgery in the 1990s; the acellular dermal matrix (ADM) originates from human skin obtained from tissue banks [119]. Through processing, a non-vital graft is obtained, providing structure and support for angiogenesis and cellular migration from the recipient site [119]. Studies have shown that ADM is an acellular and non-immunogenic structure that functions as a scaffold for host cells [120]. The acellular dermal matrix offers color and texture stability, easy handling, and uniform thickness while also reducing procedure morbidity and surgical time [121,122]. Although clinical studies have shown good results, as a biomaterial of human origin, the commercial acellular dermal matrix is known as AlloDerm^®^ (LifeCell Corporation, Branchburg, NJ, USA) [121]. Despite reducing donor site morbidity, this biomaterial has some disadvantages and limitations, such as high cost, higher resorption compared to autogenous grafts, variable integration, risk of immunogenicity, and reduced final volume [123]. In some countries, such as Brazil, AlloDerm^®^ has been suspended by the Brazilian Health Regulatory Agency (ANVISA) since 2004. The availability of this product depends on local regulatory approvals and the import policies of each region. Therefore, the best alternative to autogenous grafts is heterogeneous mucosal substitutes.

### 5.3. Heterogenous Mucosal Substitutes

The heterogeneous mucosal substitutes are mainly indicated for large grafting areas where autogenous grafting has limited availability, mostly of bovine and porcine origin [124]. The collagen matrix, composed of types I and III, is processed to ensure collagen purification without damaging its structure and is responsible for guiding cellular migration from adjacent tissues [125]. The tridimensional absorbable matrix consists of a dense collagen layer that protects against bacterial contamination and is designed for the oral cavity, allowing for matrix exposure with open healing [124]. Some available collagen matrix options on the market include Mucograft^®^ and Fibro-Gide^®^ (Geistlich Pharma, Wolhusen, Switzerland) and Mucoderm^®^ (Straumann, Basel, Switzerland). In general, these matrices function as a three-dimensional tissue scaffold and possess elastic properties that allow for better suture accommodation on-site [126]. The matrix layer facing the host is porous and spongy, providing an excellent structure for blood vessel penetration [124,125]. This characteristic promotes rapid revascularization, clot formation, endothelial cell growth, cellular differentiation, and tissue integration [125]. The degradation process of the matrix occurs gradually, depending on the size of the periodontal defect [126]. The literature recommends the use of collagen matrices for the treatment of RT1-type gingival recessions, preferably multiple, with a minimum band of keratinized tissue between 1.5 mm and 2 mm [127]. However, the use of matrix peri-implant soft tissue augmentation has shown inferior results compared to autogenous grafts [128].

In the early 1990s, Lars Hammarström in Sweden discovered enamel matrix proteins (EMPs) secreted by the epithelium of Hertwig’s sheath, which is capable of promoting periodontal regeneration [129]. Amelogenins are the main component of enamel-derived matrix proteins [130]. These proteins aggregate into supramolecular structures that form an insoluble extracellular matrix (ECM) and control the organization of developing enamel crystals [130]. In general, EMPs play a crucial role in the formation of root cementum, promoting the proliferation of mesenchymal cells, periodontal ligament fibroblasts, and osteoblasts [131]. Additionally, EMPs play a significant role in wound healing by supporting soft tissue regeneration and angiogenic activity [132]. The EMPs used in regenerative therapy are extracted from the developing embryonic enamel of porcine origin; these proteins are commercially available in the form of Emdogain^®^ gel (Straumann, Basel, Switzerland). Before the application, root surface conditioning with 24% EDTA (PrefGel^®^ Straumann, Basel, Switzerland) is required to form a natural extracellular matrix through precipitation on the root surface, stimulating essential cells for periodontal regeneration and healing [133,134].

## 6. Challenges, Innovations, and Future Perspectives

Biomaterials, including membranes, bone substitutes, and mucosal substitutes, play a fundamental role in tissue and bone regeneration by providing structural stability and promoting biological integration [135]. Tissue bioengineering is an interdisciplinary field that incorporates principles of biology, engineering, and technology to develop innovations or enhance the properties of commercially available biomaterials, aiming to optimize the regeneration process and achieve better clinical outcomes [135].

Recent in vitro studies have investigated promising methods for tissue regeneration. For example, electrical stimulation and piezoelectric ceramics promoted calcium ion flow and increased the mRNA expression of neuronal markers such as MAP2, in addition to stimulating cell proliferation and differentiation. Antibacterial effects against *E. coli* and *S. aureus* were also observed [136,137].

The challenges in GTR and GBR involve improving biocompatibility [138,139] and the bioactivity of biomaterials, which can induce immune reactivity and inflammation [140]. Non-resorbable membranes have a risk of early exposure, making them susceptible to bacterial colonization that can compromise regeneration [141]. Therefore, antibacterial-coated membranes have been tested [142,143,144]. Additionally, absorbable cross-linked membranes were developed to provide a slower degradation rate, ensuring controlled stability over the regenerative procedure [145]. A retrospective clinical study indicated that GBR using resorbable membranes simultaneously with implant placement may be a suitable clinical approach and suggested that horizontal bone reconstructions should be limited to 3 mm in order to avoid complications and obtain long-term results [146]. Furthermore, a recent study in rats tested hyaluronic acid associated with a collagen membrane and showed a delayed degradation rate due to the inhibition of macrophage infiltration [147]. In this context, various commercial synthetic bone substitutes contain different proportions of HA and β-TCP to regulate the intended resorption rate [148]. Finally, photobiomodulation has also been shown to reduce inflammation and accelerate bone tissue healing when combined with calcium hydroxyapatite (CaHA) [149].

All types of biomaterials are osteoconductive, which serve as a scaffold for bone growth, and the challenge is to enhance their performance as well as autogenous grafts [150]. To achieve this, studies aim to stimulate properties such as osteoinduction, in which growth factors, particularly bone morphogenetic protein 2 (BMP-2), promote cell differentiation into osteoblasts [150,151,152]. Another challenge is the development of personalized biomaterials, considering that systemic conditions such as diabetes [153], hypertension [154], and osteoporosis [155] are prevalent in the population and may interfere with the process of regeneration and compromise clinical outcomes. For this, one option is functionalizing biomaterials with biomolecules to improve their biological properties, such as phytotherapeutics [156,157], bone anabolic drugs [158,159], and polymers [160,161]. Similarly, studies have shown promising results with hydrogels and exosomes as scaffolds for delivering biomolecules in GTR and GBR [162,163].

Mesenchymal stem cells (MSCs) have great potential to enhance periodontal regeneration and bone formation [164]. Preclinical studies have shown that MSCs derived from the periodontal ligament have significant potential for treating intraosseous periodontal defects without significant adverse effects [165]. However, the host response and immunological safety are crucial for long-term success [166]. Moreover, the quality of transplanted stem cells and appropriate scaffolds are essential for the regeneration procedure, ensuring a safe and contaminant-free surgical delivery method [167]. The transition of MSC-based therapies from the preclinical stage to clinical application has challenges, particularly in terms of regulatory requirements and scalability. For instance, cell therapy validation is still required through multicenter, randomized, and controlled studies to confirm the long-term safety and efficacy of these therapies in applications for GTR and GBR. Regulatory approval is also complicated by differing standards among health agencies worldwide, which hinders global implementation. Furthermore, one of the main barriers to clinical use is the limited cost-effectiveness of MSC-based therapies, as large-scale production under good manufacturing practices involves high costs and complex logistics, compromising their economic feasibility [168].

Platelet aggregates are a technique already used in clinical practice to stimulate wound healing and improve the regeneration process, offering new perspectives for the treatment of periodontal and bone defects [169]. Leukocyte and platelet-rich fibrin (L-PRF) is a superior alternative to platelet-rich fibrin (PRF) due to the absence of anticoagulants, which contribute to enhanced regenerative properties [170]. L-PRF contains many growth factors and cytokines, including vascular endothelial growth factor (VEGF), transforming growth factor beta-1 (TGF-β1), platelet-derived growth factor (PDGF), fibroblast growth factor (FGF), insulin-like growth factor-1 (IGF-1), interleukins (IL-4 and IL-1β), and tumor necrosis factor-alpha (TNF-α) [171]. Therefore, L-PRF combined with bone substitutes accelerates the regenerative process [172,173].

Another innovation is manufacturing technologies, such as three-dimensional (3D) printing, which have shown great potential in producing synthetic membranes and other personalized biomaterials [174,175], allowing for the creation of grafts tailored to each patient. Current 3D printing technologies have advanced in developing scaffolds for the reconstruction of complex maxillofacial defects with essential mechanical and biological requirements. These scaffolds feature controlled interconnected porosity that supports cell infiltration and vascularization, which are critical for bone regeneration. Additionally, fixation devices produced through 3D printing offer a patient-specific alternative to standard off-the-shelf options, with the potential to decrease surgical time and enhance anatomical fit. Comparable advantages have also been observed with 3D printed anatomical models and surgical guides used in preoperative planning or during surgery [176]. However, for this approach to be widely implemented in clinical practice, it must become more accessible and efficient for large-scale production.

Currently, the focus is on developing effective solutions to improve GTR and GBR aligned with clinical needs. Future perspectives include the development of smart delivery biomaterials with growth factors or biomolecules according to tissue needs [177]. A review indicates the potential advancements in stability and resistance to degradation offered by the innovative injectable albumin platelet-rich fibrin (Alb-PRF/e-PRF) technology. These improvements suggest its promising future as a substitute for collagen membranes in various clinical applications, such as GBR, extraction site care, lateral sinus closure, and recession treatment [178]. Another promising approach is epigenetics, which allows for the modulation of gene expression associated with tissue and bone regeneration [179]. Moreover, the development of synthetic biomaterials with essential biological properties has become an established trend in tissue engineering. In addition, studies on biodegradable materials of natural origin, such as chitosan [180], cellulose [181], and silk fibroin [182] have shown good results and can be another alternative. However, many of these innovations are still in vitro or preclinical tests. To implement these advancements in clinical practice, barriers such as high production costs, clinical feasibility, and complex handling must be overcome, ensuring that new biomaterials are not only innovative but also accessible and viable for large-scale production. Additionally, maintaining the long-term functionality of regenerated tissues is crucial for clinical success.

This narrative review, although useful for providing an overview of the GTR and GBR topic, presents inherent methodological limitations. The main limitations include the absence of a systematic and reproducible search strategy, as well as the lack of risk of bias assessment for the studies included in the review. For these reasons, the performance of systematic reviews is recommended in areas that require further investigation in order to obtain more robust scientific-based evidence.

## 7. Conclusions

The use of biomaterials for GTR and GBR provides a reduction in postoperative morbidity, as it contributes to less invasive clinical procedures, serving as an alternative to autogenous grafts. Moreover, randomized clinical trials (RCTs) and systematic reviews are essential for the evaluation of new biomaterials. These studies provide more robust evidence and help guide clinical practice in the selection of safer and more effective biomaterials, allowing for the personalization of treatment protocols for each patient.

## Figures and Tables

**Table 1:** Commercially available non-absorbable membranes.

	Commercial Name	Manufacturer	Composition	Reference
e-PTFE	Gore-Tex^®^	W.L. Gore & Associates	Expanded polytetrafluoroethylene	[22]
	TefGen^®^	Geistlich Pharma		[22]
d-PTFE	Cytoplast^®^	Osteogenics Biomedical	High-density polytetrafluoroethylen	[22]
	Permamem^®^	Botiss Biomaterials		[22]
	OsseoGuard^®^	Zimmer Biomet		[22]
Ti-PTFE	Cytoplast^®^ Ti-150	Osteogenics Biomedical	Polytetrafluoroethylene + titanium	[22]
	Cytoflex^®^ Ti-reinforced	Unicare Biomedical		[22]
	OpenTex^®^-TR	Purgo Biologics		[24]

**Table 2:** Commercially available collagen-based membranes.

	Commercial Name	Manufacturer	Collagen Type	Collagen Source	Reference
Cross-linked	BioGide^®^	Geistlich Pharma	I and III	Porcine	[46]
	OSSIX Plus^®^	Dentsply Sirona	I	Porcine	[46]
	OsseoGuard^®^	Zimmer Biomet	I	Bovine	[47]
Non-cross-linked	CollaTape^®^	Zimmer Biomet	I	Bovine	[29]
	Jason^®^	Straumann	I and III	Porcine	[48]
	Mucograft^®^	Geistlich Pharma	I and III	Porcine	[49]

**Table 3:** Commercially available synthetic membranes.

Commercial Name	Manufacturer	Composition	Reference
Plenum^®^ Guide	Plenum	Polydioxanone	[52]
Resolut Adapt^®^	W.L. Gore & Associates	Poly-d,l-lactide and co-glycolide	[53]
Guidor^®^	Sunstar Americas	Poly-d,l-lactide and Poly-l-lactide blended with Acetyl tri-n-butyl Citrate	[53]
Epi-Guide^®^	Curasan Inc.	Poly-d,l-lactic acid	[53]
Vivosorb^®^	Polyganics	Poly(d,l-lactide-ɛ-caprolactone)	[53]

**Table 4:** Commercially available heterogenous bone substitutes.

Commercial Name	Manufacturer	Deproteinized Bone Origin	Reabsorption Rate	Reference
Bio-Oss^®^	Geistlich Pharma	Bovine	5–10 years	[84]
Cerabone^®^	Botiss Biomaterials	Bovine	±10 years	[85]
Endobon^®^	Zimmer Biomet	Bovine	±10 years	[86]
OsteoBiol^®^ Equimatrix	Tecnoss	Equine	6–12 months	[87]
OsteoBiol^®^ Gen-Os	Tecnoss	Porcine	4–6 months	[87]

## Data Availability

Not applicable.
